# Smoke, alcohol and drug addiction and male fertility

**DOI:** 10.1186/s12958-018-0320-7

**Published:** 2018-01-15

**Authors:** Andrea Sansone, Carla Di Dato, Cristina de Angelis, Davide Menafra, Carlotta Pozza, Rosario Pivonello, Andrea Isidori, Daniele Gianfrilli

**Affiliations:** 1 Department of Experimental Medicine, Section of Medical Pathophysiology, Food Science and Endocrinology, Sapienza - University of Rome, Viale Regina Elena 324, 00161 Rome, Italy; 2 I.O.S.& COLEMAN Srl, Naples, Italy; 3 Dipartimento di Medicina Clinica e Chirurgia, Sezione di Endocrinologia, Università “Federico II” di Napoli, Naples, Italy

## Abstract

In recent decades, the decline in human fertility has become increasingly more worrying: while therapeutic interventions might help, they are vexing for the couple and often burdened with high failure rates and costs. Prevention is the most successful approach to fertility disorders in males and females alike. We performed a literature review on three of the most common unhealthy habits – tobacco, alcohol and drug addiction – and their reported effects on male fertility. Tobacco smoking is remarkably common in most first-world countries; despite a progressive decline in the US, recent reports suggest a prevalence of more than 30% in subjects of reproductive age – a disturbing perspective, given the well-known ill-effects on reproductive and sexual function as well as general health. Alcohol consumption is often considered socially acceptable, but its negative effects on gonadal function have been consistently reported in the last 30 years. Several studies have reported a variety of negative effects on male fertility following drug abuse – a worrying phenomenon, as illicit drug consumption is on the rise, most notably in younger subjects. While evidence in these regards is still far from solid, mostly as a result of several confounding factors, it is safe to assume that cessation of tobacco smoking, alcohol consumption and recreational drug addiction might represent the best course of action for any couple trying to achieve pregnancy.

## Background

Almost 15% of all couples trying to conceive are affected by infertility, and in almost half of these cases male infertility is the sole or a contributing factor [1]. The decline of male fertility is not an empty threat: evidence points at a steadily progressive decline of sperm concentration over the past 35 years [2]. These reports have rekindled the interest in the potential impact of environmental factors and lifestyle on fertility: in order to decrease the social costs of male infertility and the resulting burdens on public health, identifying preventable factors is of the utmost importance. Sedentary behavior and obesity have both been associated with impaired male fertility [3, 4] and have often been addressed as potentially preventable factors; a more detailed review on the effects of sports and physical activity on male fertility is included in this *Special Issue*. The role of other unhealthy lifestyles, such as smoking and alcohol consumption, and environmental stressors on general health is universally recognized, but their effects on male fertility are less known [5]. Indeed, male reproductive health may be a sensitive marker of pollution [6] and environmental exposures [7, 8]. For ethical reasons, interventional studies in regards to the effects of tobacco use, second-hand smoking, recreational drug abuse and alcohol consumption are generally not feasible in humans. The vast majority of studies on these topics are therefore retrospective [8]. Sadly, this leads to a wide array of confounders, for which control is limited. A partial solution comes from animal studies; however, exposure in these models is significantly higher than in humans, and as such results should be interpreted with caution.

In order to provide an up-to-date and reliable reference in regards to the possible role of alcohol, tobacco and recreational drugs on male fertility, we performed a thorough review of existing literature and gathered all necessary data.

### Causes of male infertility

Couple infertility is defined by the failure to achieve pregnancy after at least 12 months of regular, unprotected intercourse. On the other hand, providing a fitting definition for male infertility is a more difficult task: diagnosis is traditionally based on semen analysis results, compared to the World Health Organization reference values [9].

Successful spermatogenesis is the result of the complex interaction between endocrine, paracrine and autocrine factors [10]. Unsurprisingly, several acquired and congenital conditions might impair the fine mechanisms involved in spermatogenesis (Table 1). Acquired testicular failure, as often observed following testicular torsion, orchitis, or administration of cytotoxic treatment is often associated with azoospermia. Varicocele, testicular trauma, and medications might affect fertility, although in most circumstances the spermatogenesis is impaired to a lesser extent. Genetic abnormalities, such as Klinefelter Syndrome or microdeletions in the AZF *(azoospermia factor)* region on the human male Y chromosome, usually manifest with azoospermia; however, small nucleotide polymorphisms are being investigated as a possible cause of “idiopathic” oligo-azoospermia. However, despite the recent discoveries in regards to the genetics of male infertility, to the present date most causes of oligozoospermia remain unknown.

**Table 1:** Possible acquired causes of male reproductive impairment

Hypothalamic-pituitary axis	
Compressive effect (Pituitary or brain tumors)	
Ablative effect (CNS surgery or radiotherapy)	
Infiltrative effect (fungal infections, sarcoidosis, hemochromatosis)	
Suppressive effect (exogenous AAS)	
Quantitative alterations of spermatogenesis	
Varicocele	
Previous chemotherapy or radiotherapy	
Testicular failure (torsion, orchitis, orchiectomy)	
Comorbidities (liver and kidney disease)	
Qualitative alterations of spermatogenesis	
Oxidative stress	
DNA damage	
Ductal obstructions	
Surgery (vasectomy, hernia repair, pelvic surgery)	
Neurological disorders (Multiple Sclerosis, Spinal Cord Injury, neural tube defects)	
Erectile or ejaculatory dysfunction	

*Abbreviations*: *CNS* central nervous system, *AAS* anabolic-androgenic steroid. Edited from H Tournaye, C Krausz and RD Oates [10]

Age is significantly associated with decline in semen quality, supposedly as a result of continued replications from mutated spermatogonial stem cells [11]. DNA fragmentation and chromatin condensation might also play a role in the pathogenesis of male infertility [12]. Among non-genetic causes of male infertility, oxidative stress resulting from exaggerated production of reactive oxygen species (ROS) is perhaps the most known factor. ROS are needed for capacitation, the acrosome reaction and ultimately fertilization; however, reduced clearance and excessive production are both able to induce DNA damage and defective membrane integrity in sperm cells, therefore resulting in reduced fertility potential [13]. Semen from fertile men has a more effective antioxidant capacity than that from infertile men; furthermore, immature teratozoospermic forms produce relatively more ROS than normal, mature sperm [13]. Inflammatory processes and vascular diseases, including varicocele, can supposedly increase ROS production: a thorough evaluation aimed to assess the presence of predisposing conditions is mandatory in the evaluation of any infertile male.

### Tobacco smoking and male fertility

More than 60% of noncommunicable diseases list smoking among their risk factors, and every year more than six million deaths result from tobacco consumption and second-hand smoke [14]. Despite the growing body of evidence supporting its deleterious effects, smoking is still a widespread phenomenon, as proven by recent reports from the World Health Organization. More than one-third of all male adults worldwide use tobacco [15]; similarly, approximately 30% of women of reproductive age smoke cigarettes [16]. Europe is still the leading continent in regards to tobacco use, whereas smoking rates have gradually declined in the United States in recent years [17].

### Clinical studies

The deleterious effects of smoking on fertility have been described since 1983: Olsen and colleagues identified tobacco use as one of the causes of otherwise unexplained infertility in more than 1000 females [18]. To the present date, more than 4700 different chemicals have been identified in tobacco smoke [19], ranging from heavy metals to polycyclic aromatic hydrocarbons to mutagenic chemicals. A significant association between seminal plasma lead levels and lifetime smoking estimate has been reported [20]; likewise, smoking is considered the most common source of lead and cadmium exposure [21]. Some metal micronutrients involved in the pathogenesis of oxidative stress and male infertility, including arsenic and the aforementioned cadmium and lead, are routinely inhaled during combustion of tobacco or cigarette paper [21]. These metals all have mutagenic properties and are similarly associated with increased risk of male infertility, despite no significant differences in semen volume, concentration, and motility [22, 23]. On the other hand, impairments in sperm parameters have been observed in many studies in the last decades [24, 25]: in most of them, alterations in morphology and decreased concentration, motility and viability have been observed among smokers. A significant decrease in sperm concentrations of current smokers compared with those who had never smoked has been observed in a meta-analysis of more than 2500 men from five separate studies [26]. Similarly, Kunzle and colleagues have found a significant association between smoking and reduced sperm concentration in 2100 men presenting for fertility evaluation [27].

Mechanisms resulting in impaired sperm parameters have been investigated, but definite evidence is still missing. Ultra-structural abnormalities, mostly affecting axonemal microtubules and tail alterations, have been reported in heavy smokers [28, 29]; similarly, smoking impairs the acrosome reaction [30] and capacitation [31], two processes ultimately needed for fertilization. Increased oxidative stress has been suggested as a possible mechanism resulting in impaired sperm functions. Hypoxia resulting from cigarette smoking might also be responsible for impaired spermatogenesis, even more dramatically in patients with varicocele [8, 32]. Mitochondrial activity and chromatin structure in human sperm might be impaired by several toxins, therefore negatively affecting fertilization capacity both in vivo and in vitro [17, 33, 34].

Hypothetically, chronic cigarette smoking increases liver metabolism of testosterone, while at the same time inducing secretory dysfunction of Leydig and Sertoli cells [24]. However, there seems to be no general consensus on the effects of smoking on FSH and LH production: some studies have shown lower levels of both gonadotropins among smokers, whereas different researchers have observed increased concentration of LH and/or FSH following tobacco consumption [35, 36]. Considering all the possible confounding factors, testosterone concentration is remarkably difficult to ascertain among smokers: some studies have reported increased serum testosterone and dehydroepiandrosterone levels in smokers [35, 37], whereas others have suggested that mean levels of testosterone are not significantly different [38] between smokers and non-smokers.

The American Society of Reproductive Medicine in 2012 stated that “semen parameters and results of sperm function tests are 22% poorer in smokers than in nonsmokers and the effects are dose-dependent” [16]. More recently, a meta-analysis study on a grand total of 5865 subjects has concluded that moderate and heavy smokers are more likely to have reduced sperm count and motility [17]. Evidence suggests a significant role for cigarette smoking on spermatogenesis, but on the other hand the impact of smoking on male fertility has yet to be fully elucidated. A preventive approach to infertility, suggesting smoking cessation and reduction of second-hand smoking in both women and men, should be suggested.

### Experimental studies

#### Animal studies

Cigarette smoking results in accumulation of benzo(a)pyrene (B[a]P) and cotinine, ultimately leading to DNA damage and testicular cytotoxicity in rodent models. In a recent study, Esakky and colleagues [39] reported significantly decreased expression of aryl hydrocarbon receptor (*Ahr*), and enhanced expression of Fas, FasL, BCL2, and activated caspase-3 proteins in testes exposed to cigarette smoke condensate. The reduced expression of Ahr increases susceptibility of germ cells to polycyclic aromatic hydrocarbons, while the remaining proteins all induce apoptosis via extrinsic (FAS, FASL) or mitochondrial processes (BCL, caspase-3). Tobacco use is also closely associated to reduced antioxidant activity, therefore worsening the effects of oxidative stress [40].

Exposure to cigarette smoke also impairs the activity of sorbitol dehydrogenase and lactate dehydrogenase, reflecting the effects on spermatogenesis and sperm maturation in rats [41]. Most importantly, histomorphological alterations of testes, significantly increased abnormalities in epidydimal spermatozoa and sperm DNA damage have been observed in rats exposed to cigarette smoke [41, 42]. In both in vitro and in vivo studies, nicotine resulted involved in direct impairment of sperm motility and in apoptosis induction in rat Leydig cells [43]. Mice exposed to cigarette smoke also underwent alterations in cell signal pathway networks, including ERK1/2, nuclear factor-κB and several protein kinases involved in spermatogenesis; furthermore, modified DNA methylation patterns were observed near transcriptional start sites for the *PEBP1* gene. Expression of *PEBP1* results in production of phosphatidylethanolamine-binding protein 1, a protein that in humans has been shown to interact with C-Raf, MAP2K1 and MAPK1 [44].

High doses of nicotine induce a significant decrease in sperm count and motility in prepubertal and adult rats exposed to progressively increasing concentrations of nicotine [45]. Impaired testicular function is also reflected in significantly decreased testosterone levels [45, 46], although as previously suggested it’s still unclear whether these findings are valid for humans as well as rodents.

Smoking cessation, on the other hand, improves indices of sexual health for long-term male smokers [47] and, based on finding in animal models, might improve sperm parameters [48]. However, to the present date, no conclusive evidence on the real improvements in male fertility following smoking cessation has been obtained.

#### Studies in humans

Recent genome-wide studies have identified alterations in the methylation profile of 95 sites in smokers [49]. Smoking-related DNA damage and methylation patterns are observed in several human tissues - even those who are not directly exposed to it because of indirect systemic exposure [50]. DNA adducts and DNA damage are inversely associated with sperm parameters, mostly concentration and motility, and both are transmitted to the zygote with little chance of repair by the ovum [51]. Sperm DNA fragmentation is also linked with increased rates of spontaneous abortion [52] and should therefore be carefully assessed in subjects undergoing assisted reproduction techniques. Oxidative DNA damage [53] and higher cadmium levels [54] resulting from tobacco consumption are similarly associated to impaired fertility, resulting in longer time to pregnancy for couples.

Smoking also reduces sperm creatine kinase activity, therefore impairing sperm motility and energy homeostasis [55]. In vitro studies have shown nicotine, cotinine and cadmium as possible culprits; in vivo, both smoking duration and amount of cigarettes smoked per day seem able to reduce creatine kinase activity in sperm [56, 57].

### Alcohol consumption and male fertility

Clinical and experimental studies have examined alcohol consumption as a potential risk factor for male infertility, exerting a direct effect on both testosterone metabolism and spermatogenesis.

### Clinical studies

The link between alcohol and fertility was investigated in 1985 for the first time. The analysis of seminal fluid samples and the hormonal evaluation of 20 men with alcohol dependence syndrome revealed a significant decrease in testosterone levels, seminal fluid volume and sperm concentration in chronic alcoholics than in controls [58]. Subsequently, a prospective autopsy study showed that a significant percentage of heavy drinkers (52.3%) had partial or complete spermatogenic arrest, and that the mean testicular weight of heavy drinkers was slightly but significantly lower compared with that of controls [59]. Muthusami et al., in 2005, found in chronic alcoholics a significant increase of FSH, LH, and E2 levels, while testosterone was significantly decreased. Semen volume, sperm count, motility, and number of morphologically normal sperm were significantly decreased [60]. In 2011 one meta-analysis including 57 studies and 29,914 subjects found a significant association between alcohol, semen volume, sperm morphology and sperm motility [61].

Therefore, chronic and excessive alcohol intake seem to have a detrimental effect on male reproductive hormones and on semen quality. Conversely, the effect of moderate alcohol intake is still under debate.

A cross sectional study from Jensen et al. on 8344 healthy men suggest that moderate alcohol intake (median weekly intake 8 units) is not adversely associated with semen quality in healthy men, whereas it was associated with higher serum testosterone levels [62]. Furthermore, chronic alcohol consumption seem to influence fertility more than acute alcohol consumption. Hansen et al. evaluated the association between last 5 days of alcohol intake, semen quality and reproductive hormones in a cross-sectional study among 347 men [63]. Alcohol intake was associated with impairment of most semen characteristics but without a coherent dose–response pattern. There was a tendency towards lower semen characteristics at higher intake of alcohol past 5 days and a hormonal shift towards higher estradiol/testosterone ratio. The importance of the timing of alcohol consumption was also established from Condorelli et al. The Authors retrospectively evaluated semen and hormonal parameters of moderate alcohol users, comparing occasional drinkers with daily drinkers. Within each group, a further comparison was made between the fertile subjects (pregnancy over the past 12 months) and the infertile patients (no evidence of pregnancy or fertilisation for at least 12 months). The results showed that infertile patients belonging to group of ‘daily drinkers’ have a semen quality and hormonal characteristics significantly worse compared with the other groups [64]. Time-to-pregnancy for was significantly longer in couples in which the male partner consumed more than 20 units of alcohol on a weekly basis [65], but literature is severely lacking concerning more moderate consumption of alcoholic beverages.

The mechanisms underlying the damage of alcohol on fertility are not yet fully clarified. Close and colleagues reported that current heavy alcohol users have significantly higher leukocyte concentrations in the seminal fluid compared with nonusers. After controlling for past sexually transmitted diseases and multiple substance exposures in a multivariate model, alcohol users had only a trend towards increased leukocytes in the seminal fluid [66]. Some Authors hypothesized that also maternal alcohol consumption during pregnancy can influence semen quality in the male offspring. From a cohort of Danish pregnant women established in 1984–1987, 347 young adult sons were selected for a follow-up study conducted in 2005–2006. The results of this study showed that the sperm concentration decreased with increasing prenatal alcohol exposure. No associations were found for sperm motility, sperm morphology, or any of the reproductive hormones, including testosterone [67].

### Experimental studies

#### Animal studies

Alcohol consumption has often been associated with an increase in β-endorphin levels that could be involved in testicular damage, inducing sperm apoptosis. In 1999 Yin and colleagues showed that morphine induces the expression of the protein Fas (also known as CD95 or APO-1), a receptor on the cell surface that triggers the cell’s suicide by apoptosis when it binds to its ligand, FasL [68]. Furthermore, an experimental study demonstrated that treatment with naloxone and naltrexone in adult and pubertal male rats could prevent alcohol-induced testosterone inhibition [69].

Apoptosis is one of the responsible factors for spermatozoal chromatin disorders. Several studies showed that ethanol consumption disturb nuclear maturity and DNA integrity of spermatozoa. Talebi and colleagues evaluated the effect of ethanol consumption on sperm parameters and chromatin integrity of spermatozoa aspirated from cauda epididymis of rats. Results revealed that sperm progressive and non progressive motility of ethanol-consuming rats were significantly decreased compared with control animals and an alteration of nuclear maturity and DNA integrity [70].

Therefore, spermatogenic cells undergo apoptosis when treated with ethanol but the mechanism remain unclear. In the study of Jana and colleagues, intra-peritoneal injection of ethanol induced apoptotic spermatogenic cell death with a decrease in the plasma and intra-testicular testosterone in adult male mice. In this study, western blot analysis revealed that repeated ethanol treatment decreased the expression of steroidogenic acute regulatory protein (StAR), 3b-hydroxysteroid dehydrogenase (3b-HSD) and 17b-hydroxysteroid dehydrogenase (17b-HSD); increased the expression of active caspase-3, p53, Fas and Fas-L; and led to up-regulation of Bax/Bcl-2 ratio and translocation of cytochrome c from mitochondria to cytosol in testis. Moreover, repeated ethanol treatment led to upregulation of caspase-3, p53, Fas and Fas-L transcripts; increase in caspase-3 and caspase-8 activities; diminution of 3b-HSD, 17b-HSD and GPx activities; decrease in the mitochondrial membrane potential along with ROS generation and depletion of glutathione pool in the testicular tissue [71].

#### Studies in humans

Polycyclic aromatic hydrocarbons (PAH) are ubiquitous pollutants in the environment, which are able to form DNA adducts when they are activated to DNA reactive metabolites. Measurement of DNA adducts is a widely used marker of DNA damage induced by environmental pollutants. Gaspari and colleagues evaluated data on (PAH)-DNA adducts in 182 men with morphological abnormalities in the sperm, finding a significant negative association was between daily alcohol consumption and PAH-DNA adducts in sperm [72]; similarly, Rossi and colleagues reported that increased alcohol consumption was associated with fertilization failure and reduced live birth rates [73], with a 21% decline in couples in which both partners drank more than 4 units per week. On the contrary, in the study of Horak et al., no correlation between alcohol and sperm DNA adducts was found [74]. Finally, Loft evaluated the level of oxidative DNA damage in terms of 7-hydro-8-oxo-20-deoxyguanosine (8-oxodG) in sperm DNA among 225 first-pregnancy planners and the 8-oxodG level was not significantly associated with consumption of alcohol [53].

Finally, the genetic background may modulate the impact of alcohol on spermatogenesis. The glutathione S-transferase (GST)-M1 genotype may be associated with a greater susceptibility to develop, via direct mechanism at testicular level, alcohol-induced spermatogenesis disorders. An autopsy study comprising 271 subjects showed that among 50 moderate drinking men, 48% had partial and 10% complete spermatogenic arrest. Among the 21 men with normal spermatogenesis, 42.9% had GST M1 genotype with a frequency similar to that found in men with partial or complete spermatogenic arrest (44.8%). Among the 212 heavy-drinking men, 21.2% of the subjects had normal spermatogenesis, 36.3% had partial spermatogenic arrest, 38.2% showed complete arrest spermatogenic arrest and 4.2% showed Sertoli cell-only syndrome. Interestingly, 27 of the 45 heavy drinkers with normal spermatogenesis (60%) men had the GST M1 genotype. The finding that >20% of heavy drinkers had normal spermatogenesis suggests that the GST M1 genotype exerts a protective effects on alcohol-induced spermatogenesis disorders [75].

### Drugs addiction and male fertility

Up to almost one in four men under the age of 35 years uses recreational drugs [76]. Several studies have suggested that these drugs might have adverse effects on human reproduction. Cannabis smoking has been shown to negatively impact male fertility, with an effect on hypothalamus-pituitary-gonadal axis, spermatogenesis, and sperm function, as cannabinoid receptors are expressed in the anterior pituitary, Leydig cells, Sertoli cells and in testicular tissues [77]. Similarly, negative effects on male fertility have been reported in subjects using cocaine, MDMA (ecstasy) and opioids. Cocaine use has been associated with other high-risk behaviors, such as tobacco smoking and sexually transmitted diseases, and might lead to testicular cell apoptosis; opioids act on the HPG axis, possibly resulting in hypogonadal hypogonadism; DNA damage and tubular degeneration have been described in rats treated with MDMA. Prevention of male infertility might be achieved by identifying and addressing the consequences of the “illicit drug epidemic”.

### Clinical studies

In literature, human data on hormone levels following marijuana exposure are conflicting. Referring to animal studies it could be possible that consuming cannabis decrease serum luteinizing hormone and testosterone levels, but in clinical studies a univocal interpretation is lacking.

A study from Kolodny et al. on 20 men who used marijuana chronically showed significantly lower levels of plasma testosterone in this group than that in the control-group. Decreased testosterone was dose related. Abstention from marijuana use or stimulation with human chorionic gonadotropin during continued marihuana use produced marked increases in testosterone [78]*.* These results have been later contradicted. In 66 males neither chronic nor acute intake of marijuana had a significant effect on plasma testosterone levels, but also subjects who drank cannabis as a tea were included [79]*.* In a study of 27 men, no statistically significant changes in plasma testosterone levels were observed during and after the smoking period as compared with the pre-smoking base-line levels [80]. In four healthy males subjects, cannabis smoking significantly depressed plasma LH, while cortisol significantly increased [81]. On the contrary, in the study from Gundersen et al. on 1215 Danish healthy young men, of whom 45% had smoked marijuana during the past 3 months, marijuana use was associated with increased serum testosterone to the same level as cigarette smoking [82].

Conversely, regarding the effect of cannabis on spermatogenesis, clinical studies showed an effect on volume, number morphology, motility, and fertilization capacity.

In the study from Gundersen et al. regular use of marijuana was found to be associated with an impairment in semen quality, while irregular use seems to be irrelevant [82]. A study from Hembree et al. showed an association between marijuana use and decreased sperm count, which persisted in the following 4-week recovery period [83]. These data were confirmed also by a case study on a multidrug addict, in which semen abnormalities were detected before and 2 years after cessation of the abuse [84]*.* In a recent unmatched case-referent study with 1700 participants, it was clearly reported that cannabis exposure is a risk factor for poor sperm morphology [85]. Only one study on 159 men attending an infertility clinic demonstrated a positive correlation between marijuana use and percentage of motile sperm [66].

There are no studies on the effect of cannabis on the reproductive organs of men. Only Kolodny et al. reported no change in testicular size and texture in chronic marijuana users [78]. The differing results of these reports may in part be due to study design, the ingestion of other pharmacological agents, such as narcotics, alcohol and cigarette smoking.

Cocaine intake during pregnancy severely affects fetal development; however, little is known in regards to its effects on male fertility. The same applies to MDMA (Ecstasy): animal models might help understand the specific effects of both substances on male fertility. Bracken et al. [86] reported increased use of cocaine among subjects with lower sperm counts and motility; Samplaski et al. [87] more recently suggested that the higher rates of concurrent substance abuse, tobacco use and infections among cocaine users might lead to biased results.

Opioids act on the hypothalamic-pituitary axis by inhibiting the pulsatility of GnRH secretion: the resulting suppression of FSH and LH release consequently leads to impaired spermatogenesis and reduced testosterone concentrations [76]. Vuong et al. [88] performed a lengthy review of the effects of opioids on endocrine parameters, and concluded that there is still insufficient information on the long-term effects of opioids in regards to fertility despite concrete evidence of opioid-induced hypogonadism. Recent reports suggest that both sperm concentration and quality are impaired in opioid abusers: increased rates of DNA fragmentation and reduced expression of catalase-like and superoxide dismutase-like activity were observed in addict men compared to age-matched healthy volunteers [89].

### Experimental studies

#### Animal studies

Acute treatments with cannabinoids can decrease the fertilising capacity of sea urchin sperm [90]. In rodent studies, high THC doses caused a modest increase in abnormally formed sperm. Moreover, long-term cannabinoid exposure in male mice disrupted spermatogenesis and induced aberrations in sperm morphology [91].

Wenger and colleagues showed that THC alters pituitary LH release by inhibiting the release of LHRH, injecting THC into the third cerebral ventricle of male rats [92]. Smith et al. [93] found a significant decrease in serum testosterone concentration following acute doses of THC in rhesus monkeys. A study on adult male mice showed regressive changes in the testes and suppressed sperm count, viability and motility, caused by chronic intake of bhang. Bhang intake also caused significant decline in circulating testosterone level due to decline in testicular 3b HSD enzyme activity, a significant variation in the CB1 and CB2 receptors and FAAH protein levels (Fatty Acid Amide Hydrolase) in testes of mice exposed to bhang [94]. Finally, repeated subcutaneous administration of cannabis extract and delta-9-tetrahydrocannabinol reduced significantly fructose and citric acid contents of male reproductive organs of prepubertal as well as adult albino rats in a dose-related manner in the testis, prostate as well as in the epididymis [95].

Effects of cocaine and MDMA on fertility have been evaluated in animal models: in rodents, cocaine is able to induce prolonged vasoconstriction of testicular blood vessels, resulting in ischemic and reperfusion injuries [96]. In 1996, George et al. reported that in rats long-term exposure to cocaine resulted in reduced diameter of seminiferous tubules and similarly decreased number of total germ cells [97]. More recently, increased oxidative stress has been observed in mice following chronic administration of cocaine [98], suggesting a possible mechanism for testicular damage. Similar findings have been reported for Ecstasy and opioids: Barenys et al. described significantly decreased sperm concentration and motility, together with increased rates of DNA damage and tubular degeneration, in rats treated with varying dosages of MDMA [99], and similar findings have been described in mice treated with either tramadol [100] or morphine [101].

#### Studies in humans

Whan et al. investigated the effects of delta-9-tetrahydrocannabinol (THC) on human sperm function in vitro. Both therapeutic and recreational levels of THC determined a dose dependent reduction of sperm motility and of spontaneous acrosome reactions [102]. Subsequent studies confirmed these results [103], but the mechanism still remained not fully understood. Morgan et al. investigated the effects of WIN 55,212-2, a CB1 cannabinoid receptor agonist, and D9-tetrahydracannabinol (D9-THC) on the ATP levels and motility of murine sperm in vitro. High concentrations of WIN 55,212-2 or D9-THC inhibit ATP production in sperm; this effect of WIN 55,212-2 is CB1 receptor dependent whereas that of D9-THC is not [104].

## Conclusions

**Fig. 1:**
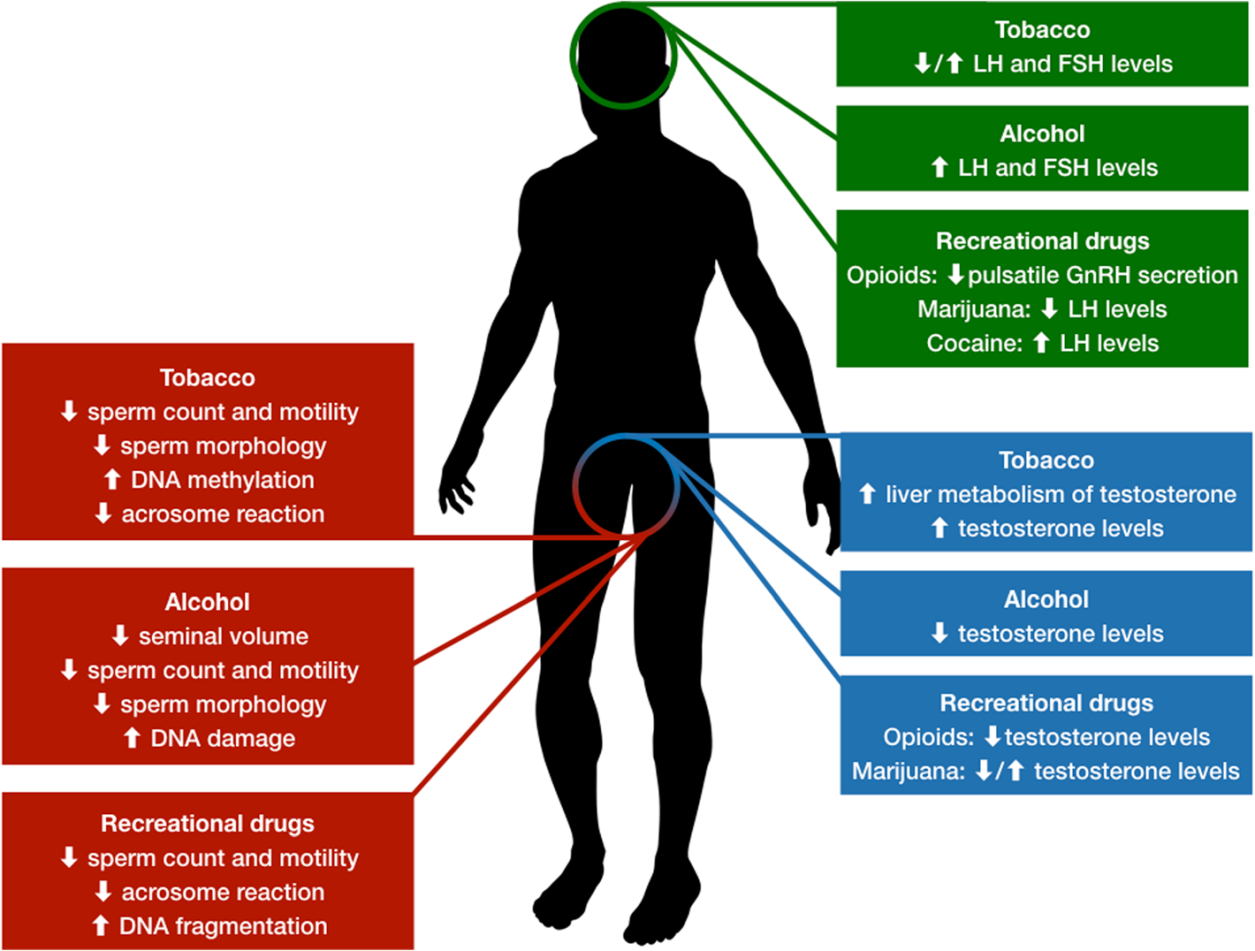
Effects of smoking, alcohol and drug abuse on spermatogenesis (left) and hormonal parameters (right) in males

Despite the lack of solid evidence from interventional studies, which are for the most part not feasible in humans, it is clear that smoking, alcohol use and recreational drug consumption are somehow able to impair male fertility, with possible synergistic, rather than addictive, effects. Impairments in spermatogenesis and sperm parameters as well as increased DNA methylation and oxidative stress have been observed in humans and animal models alike; similarly, effects on endocrine control of reproductive and sexual function have been reported in clinical and experimental studies (Tables 2 and 3, Fig. 1). Discontinuation of all these habits should be suggested in all patients undergoing investigation for infertility in order to provide the best outcomes, although little is known in regards to the time needed for cessation of negative effects.

**Table 2:** Effects of tobacco smoking, alcohol consumption and drug addiction on spermatogenesis and sperm parameters

	Studies in humans	Animal studies
Tobacco smoking	Reduction of sperm concentration [26, 27] Impairment of sperm motility [17] Increase in ultra-structural abnormalities (axonemal microtubules and tail alterations) [28, 29] Impairment of mitochondrial activity and chromatin structure [17, 33, 34] Impairment of acrosome reaction and capacitation [30, 31] Increase of DNA methylation patterns [49] Alterations in ERK1/2, nuclear factor-κB signal pathway [44] Reduction of creatine kinase activity in sperm [55–57]	Increase in apoptosis via extrinsic (FAS, FASL) or mitochondrial processes (BCL, caspase-3) in rats [39, 40] Reduction of antioxidant activity in rats [40] Increase in DNA damage in sperm rats [41, 42]; of DNA methylation patterns *PEBP1* gene in rats [44] Reduction of sperm count and motility in rats [45]
Alcohol consumption	Reduction of seminal volume and sperm concentration [58, 61, 62] Impairment of sperm motility and morphology [60, 61] Increased sperm polycyclic aromatic hydrocarbon DNA adducts [72]	Increase in apoptosis (by upregulation of Fas) of spermatogenic cells in rats [68] Alteration of nuclear maturity and DNA integrity in sperm cells of rats [70] Increase in ROS generation and depletion of glutathione pool in testicular tissue of rats [71] Impairment of sperm motility in rats [70]
Drug addiction		
Marijuana	Decrease in sperm count [82–84] Impairment of sperm morphology and motility [85, 102, 103] Reduction of spontaneous acrosome reactions [102, 103]	Impaired sperm concentration and morphology in rats [91] Inhibition of sperm ATP production in rats [104] Reduction of fructose and citric acid contents in testis and prostate of male rats [95]
Cocaine	Impairment of sperm counts and motility [86]	Vasoconstriction of testicular blood vessels in rats resulting in ischemic and reperfusion injuries [96] Reduction of diameter of seminiferous tubules [97] Decrease in number of total germ cells [97] Increase in oxidative stress in mice [98]
Opioids	Impaired spermatogenesis [76] Increased rates of DNA fragmentation in humans [89] Reduced expression of catalase-like and superoxide dismutase-like activity [89]	Increase of rates of DNA damage and tubular degeneration in rats [100, 101]
Amphetamines and Ecstasy	No data available	Vasoconstriction of testicular blood vessels in rats [96] Increase in rates of DNA damage and tubular degeneration in rats [99]

**Table 3:** Effects of tobacco smoking, alcohol consumption and drug addiction on hormone levels

	Studies in humans	Animal studies
Tobacco smoking	Increase in liver metabolism of testosterone [24] Reduction of LH and/or FSH levels [34] Increase in concentration of LH and/or FSH [35] Increase in serum testosterone and DHEA levels [35, 37]	Reduction of testosterone levels in rats [45, 46]
Alcohol consumption	Decrease in testosterone levels [58, 59] Increase in FSH, LH, and E2 levels [60]	Decrease in plasma and intra-testicular testosterone in rats [71]
Drug addiction		
Marijuana	Decrease in levels of plasma testosterone [78] Decreased levels of plasma LH, and increased levels of plasma cortisol [81] Increased serum testosterone [82]	Inhibition of LHRH release in rats [92] Decrease in testosterone levels in monkeys [93]
Cocaine	Increase in LH levels without modifying testosterone following acute administration [97]	Increase in LH levels; No effects on testosterone and estradiol in rats [97]
Opioids	Inhibition of the pulsatility of GnRH secretion; reduction of LH, FSH and testosterone levels [76]	Reduction of LH, without effect on FSH and testosterone levels in rats [101]
Amphetamines and Ecstasy	No data available	No effects on LH, FSH, Testosterone levels in male rats [99]
